# Mycovirus-Induced Tenuazonic Acid Production in a Rice Blast Fungus *Magnaporthe oryzae*

**DOI:** 10.3389/fmicb.2020.01641

**Published:** 2020-07-17

**Authors:** Akihiro Ninomiya, Syun-ichi Urayama, Rei Suo, Shiro Itoi, Shin-ichi Fuji, Hiromitsu Moriyama, Daisuke Hagiwara

**Affiliations:** ^1^Faculty of Life and Environmental Sciences, University of Tsukuba, Tsukuba, Japan; ^2^Microbiology Research Center for Sustainability, University of Tsukuba, Tsukuba, Japan; ^3^College of Bioresource Sciences, Nihon University, Fujisawa, Japan; ^4^Faculty of Bioresource Sciences, Akita Prefectural University, Akita, Japan; ^5^Department of Applied Biological Sciences, Tokyo University of Agriculture and Technology, Fuchu, Japan

**Keywords:** mycovirus, *Magnaporthe oryzae*, secondary metabolism, mycotoxin, tenuazonic acid

## Abstract

Fungi are a rich source of natural products with biological activities. In this study, we evaluated viral effects on secondary metabolism of the rice blast fungus *Magnaporthe oryzae* using an isolate of APU10-199A co-infected with three types of mycoviruses: a totivirus, a chrysovirus, and a partitivirus. Comparison of the secondary metabolite profile of APU10-199A with that of the strain lacking the totivirus and chrysovirus showed that a mycotoxin tenuazonic (TeA) acid was produced in a manner dependent on the mycoviruses. Virus reinfection experiments verified that TeA production was dependent on the totivirus. Quantitative reverse transcription PCR and RNA-sequencing analysis indicated the regulatory mechanism underlying viral induction of TeA: the totivirus activates the TeA synthetase gene *TAS1* by upregulating the transcription of the gene encoding a Zn(II)_2_-Cys_6_-type transcription factor, *TAS2*. To our knowledge, this is the first report that confirmed mycovirus-associated regulation of secondary metabolism at a transcriptional level by viral reinfection. Because only treatment with dimethyl sulfoxide has been reported to trigger TeA production in this fungus without gene manipulation, our finding highlights the potential of mycoviruses as an epigenomic regulator of fungal secondary metabolism.

## Introduction

Fungi produce structurally diverse secondary metabolites (SMs), and thus are a rich source of compounds for drug discovery. Since discovery of the first antibiotic penicillin, thousands of bioactive compounds, such as cyclosporine and lovastatin, have been found in fungi and reported (Bérdy, 2005). In contrast, some fungi can produce mycotoxins, which are a great threat to human health. For example, aflatoxins, carcinogenic mycotoxins produced by certain *Aspergillus* species, cause poisoning of grains (Bennett and Klich, 2003). A human fungal pathogen, *Aspergillus fumigatus*, produces a mycotoxin gliotoxin, which is implicated in the virulence of the fungus (Sugui et al., 2007). Studies of fungal genomes have revealed that fungi have the potential to produce more SMs than expected, as they have a large number of genes for secondary metabolism (Khaldi et al., 2010; Sanchez et al., 2012). To broaden the fungal chemical space, several studies have attempted to trigger the silent secondary metabolism (Brakhage and Schroeckh, 2011).

As is often the case with bacteria, fungi are infected with viruses called mycoviruses. Unlike bacterial phages, mycoviruses do not lyse the cells of their host but are transmitted intracellularly, and they are thus considered to behave as a symbiont. Most of the known mycoviruses are RNA viruses with an RNA-dependent RNA polymerase (RdRp) encoded in their genome, which is used for phylogenetic classification of mycoviruses. In the history of mycovirus research, the influence of mycoviruses on plant pathogenic fungi have been intensively analyzed because such viruses have potential as pest control agents. For example, cryphonectria hypovirus 1 reduces the pathogenicity of its host, a chestnut blight fungus, *Cryphonectria parasitica* (Nuss, 2005). *C*. *parasitica* harboring cryphonectria hypovirus 1 has been used for the control of chestnut blight in Europe. In addition to reduced virulence, mycoviruses cause their hosts to undergo a characteristic change of phenotype, such as colony morphology, mycelial growth, and sexual reproduction (Ghabrial et al., 2015). Thus, mycoviruses are considered to be epigenomic factors that expand the physiological diversity of their hosts.

The effect of mycoviruses on fungal secondary metabolism has been investigated in particular on mycotoxin production. In an early study, aflatoxin production by *Aspergillus flavus* is repressed by a mycovirus (Schmidt et al., 1986). Kim and co-workers revealed a decrease of trichothecene production in *Fusarium graminearum* induced by a double-stranded RNA virus (Chu et al., 2002), and Okada et al. (2018) reported chrysovirus-induced enhancement of phytotoxin production in *Alternaria alternata*. Recently, Nerva et al. (2019) reported overproduction of a carcinogenic mycotoxin, ochratoxin A (OTA) in *Aspergillus ochraceus* induced by a partitivirus. Curiously, a gene encoding polyketide synthase (PKS) for OTA biosynthesis was absent in the strain used in the study. Thus, the regulation mechanism of OTA production by partitiviruses remains unclear. So far, although some reports provided data showing that mycoviruses affect host fungal mycotoxin production, the induction mechanism has been poorly understood.

To gain more insight into mycovirus regulation of fungal secondary metabolism, more studies are needed. In a previous study, a rice blast fungus, *Magnaporthe oryzae* APU10-199A, which was co-infected with three types of viruses, a totivirus, a chrysovirus, and a partitivirus, was isolated and characterized (Higashiura et al., 2019). Although the three types of RNA viruses are widely distributed in filamentous fungi and are well studied (Ghabrial et al., 2015), the effects on secondary metabolism have been poorly understood. Here, we demonstrate totivirus-induced production of a mycotoxin tenuazonic acid (TeA) in the *M. oryzae* strain. We also report induced expression of the biosynthetic gene for TeA. To the best of our knowledge, this is the first report that indicates the molecular mechanism underlying regulation of fungal secondary metabolism by a mycovirus. We point out the potential of mycoviruses as an agent that controls fungal secondary metabolism.

## Materials and Methods

### Microorganisms and Culture Conditions

The *M. oryzae* APU10-199A was isolated from symptomatic leaves of the japonica rice cultivar Akitakomachi in Akita Prefecture, Japan in our previous work (Higashiura et al., 2019). The *M. oryzae* APU10-199A strain harbors a totivirus, chrysovirus, and partitivirus. A strain that lacks the totivirus and chrysovirus (here we named the strain APU10-199A_P) was obtained from *M*. *oryzae* APU10-199A during single conidia isolation. Co-infection of three viruses (APU10-199A) and loss of totivirus and chrysovirus (APU10-199A_P) were confirmed by gel electrophoresis of dsRNA extracted from the strains and RT-PCR in the previous research (Higashiura et al., 2019). *M*. *oryzae* strains were grown on potato dextrose agar (PDA; BD, Franklin Lakes, NJ, United States), and agar plugs were inoculated into liquid media. Liquid culture was performed using potato dextrose broth (PDB) (BD), soy sauce-sucrose (SS) medium (5% soy sauce; Shiboritate Nama Shoyu, Kikkoman, Chiba, Japan; 5% sucrose in tap water) (Nukina, 1999), or IPN medium (20% vegetable juice; Yasai Ichinichi Kore Ippon, Kagome Co., Ltd., Aichi, Japan; 0.3% CaCO_3_).

### Analysis of Secondary Metabolite Profile

The strains were cultured in 60 mL PDB, SS, and IPN media in a 200 mL Erlenmeyer flask at 25°C with agitation at 40 rpm for 2 weeks or with agitation at 150 rpm for 5 days. After filtration of the culture broth through miracloth (Merck Millipore, Burlington, MA, United States), the filtrate was extracted by an equivalent volume of ethyl acetate, which was dried *in vacuo*. The extracts were dissolved in dimethyl sulfoxide (DMSO) and analyzed using a 1260 Infinity LC system (Agilent Technologies, Inc., Santa Clara, CA, United States) with a Poroshell 120 EC-C18 column (ϕ3.0 mm × 100 mm, particle size 2.7 μm; Agilent). The high-performance liquid chromatography (HPLC) analytical condition was a gradient elution of 5–100% acetonitrile containing 0.5% acetic acid for 18 min.

### Isolation of Tenuazonic Acid

*Magnaporthe oryzae* infected with totivirus was cultured in SS medium (1.2 L) for 5–7 days at 25°C with agitation at 150 rpm. The culture supernatant was extracted with ethyl acetate, and the dried extract was subjected to octadecylsilane flash chromatography, which yielded 0, 20, 40, 60, and 100% methanol fractions. The 20 and 40% methanol fractions were purified using the above-mentioned HPLC system with a COSMOSIL 5C_18_-AR-II column (ϕ10 mm × 250 mm; Nacalai Tesque, Kyoto, Japan) to supply TeA (6.0 mg). The HPLC analytical condition was an isocratic elution of 32% acetonitrile containing 0.5% acetic acid for 20 min.

### NMR and HRESIMS Analysis

Nuclear magnetic resonance (NMR) spectra were recorded on a 500-MHz ECA500 NMR spectrometer (JEOL Ltd., Tokyo, Japan) at 293 K. The NMR chemical shifts ^1^H and ^13^C were referenced to the solvent peaks: δ_H_ 3.30 and δ_C_ 49.0 for CD_3_OD (Eurisotop, Saint-Aubin, France). High-resolution electrospray ionization mass spectrometry (HRESIMS) analysis was performed using UPLC-SYNAPT G2 HDMS (Waters, Milford, MA, United States).

*Tenuazonic acid* (compound **1**): ^1^H and ^13^C NMR data – see Supplementary Table S1; HRESIMS *m/z*: [M + H]^+^; calculated for C_10_H_16_NO_3_ 198.1130, result was 198.1127.

### qRT-PCR Analysis

*Magnaporthe oryzae* APU10-199A and APU10-199A_P were cultured for 7 days at 25°C in SS medium with agitation at 150 rpm. Mycelia were ground to a fine powder with a mortar and pestle. Total RNA was extracted by TRIzol Reagent (Thermo Fisher Scientific, Waltham, MA, United States), purified by PureLink RNA Mini Kit (Thermo Fisher), treated with DNase I (Thermo Fisher), and then purified by RNA Clean & Concentrator (Zymo Research, Irvine, CA, United States). cDNA was synthesized using ReverTra Ace qPCR RT Master Mix with gDNA Remover (TOYOBO, Osaka, Japan), and quantitative reverse transcription polymerase chain reaction (qRT-PCR) was performed using Brilliant III Ultra-Fast SYBR Green QPCR Master Mix (Agilent). The expression levels of the genes of interest were normalized against those of the β-tubulin gene. Sequences of primers used in this study are given in Supplementary Table S2.

### Reinfection of Mycovirus

Virus infection was performed via hyphal anastomosis between the virus-infected original isolate APU10-199A as a donor and APU10-199A_P as described previously (Yaegashi et al., 2011). First, the APU10-199A_P was transformed with the *hph* gene as described previously (Higashiura et al., 2019). To proceed anastomosis, the donor and recipient strains were co-inoculated with 1 cm distance on a PDA plate and incubated at 25°C for 14 days. Then, several plugs picked up from the border of two colonies were subcultured four times on PDA plates containing hygromycin B (200 μg/mL) to recover the strains derived from APU10-199A_P with *hph* gene. The recovered hygromycin-resistant strains were confirmed to harbor viruses by RT-PCR by using primers specific to each virus (for sequences of primers see Supplementary Table S2).

### RNA-Sequencing Analysis

Each *M*. *oryzae* strain was cultured for 5 days at 25°C in SS medium with agitation at 150 rpm. Total RNA was extracted and purified as described above. Novogene (Beijing, China) supported library preparation, sequencing, and partial data analysis. The reads were mapped to reference genomes of *M*. *oryzae* 70-15 (GCA_000002495.2), and the read count per gene was performed by CLC Genomics Workbench (QIAGEN, Hilden, Germany).

### Data Availability

The datasets generated for this study can be found in the DDBJ DRA under accession number SSUB014300.

## Results

### Mycoviruses Affected Tenuazonic Acid Production

**FIGURE 1:**
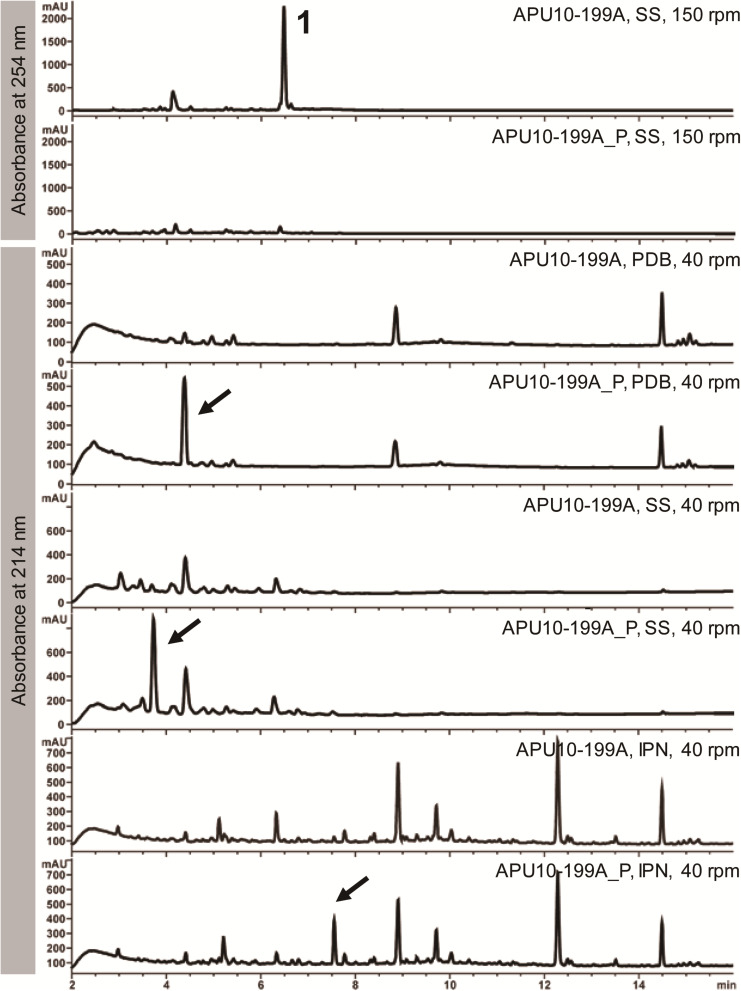
Secondary metabolite profiles of APU10-199A and APU10-199A_P. Each strain was cultured in three different media – soy sauce-sucrose (SS) medium, potato dextrose broth (PDB), and IPN medium – with agitation at two different rates (40 and 150 rpm). Production levels of the compounds shown by the arrows were higher in APU10-199A_P than in APU10-199A. These data show a representative profile from three independent culture experiments.

First, we compared the SM profiles of APU10-199A with those of APU10-199A_P, the strain lacking the totivirus and chrysovirus. Because fungal secondary metabolism is influenced by nutrient regimes and physical parameters (Bode et al., 2002), we cultured *M*. *oryzae* in three different media: SS, PDB, and IPN (for composition, see section “Materials and Methods”) with agitation at two different rates (40 and 150 rpm). As a result, we found three major peaks in the culture from APU10-199A_P, which were hardly detectable in APU10-199A when cultured at 40 rpm (Figure 1). When cultured in SS medium at 150 rpm, the production of compound **1** was observed in the culture of APU10-199A but not in that of APU10-199A_P. Metabolite analysis was not performed in PDB and IPN at 150 rpm due to their slow growth. These data showed that the production of compounds was affected by the mycoviruses. Hereinafter we focus on the virus-induced production of **1**.

**FIGURE 2:**
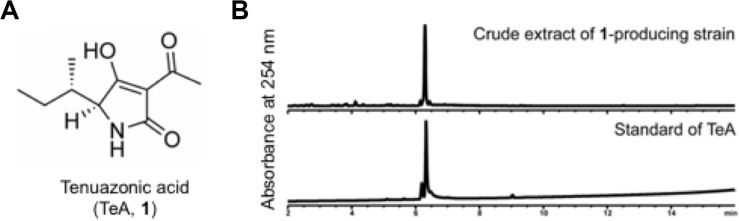
**(A)** Chemical structure of tenuazonic acid (TeA). For spectroscopic data, see the Supplementary Data. **(B)** HPLC chart of the extract of the TeA-producing strain and TeA standard.

To identify compound **1**, we cultured APU10-199A in SS medium (1.2 L) with agitation at 150 rpm, and isolated 6.0 mg of **1** from the culture supernatant. The molecular formula of **1** was determined by HRESIMS to be C_10_H_15_NO_3_ (Supplementary Figure S1). On the basis of NMR analysis (Supplementary Table S1), we assigned the planar structure of **1** as TeA (Figure 2A). Finally, we identified **1** as TeA because the chemical shifts of **1** were in good agreement with the reported value (Nolte et al., 1980), and **1** was coeluted with the TeA standard in HPLC (Figure 2B). TeA is a mycotoxin reported from several filamentous fungi (Rosett et al., 1957), such as *Alternaria* and *Magnaporthe* species. TeA inhibits protein synthesis in mammalian cells by preventing peptide bond formation (Carrasco and Vazquez, 1973).

### Mycoviruses Affected Expression of Biosynthetic and Regulator Genes for TeA

**FIGURE 3:**
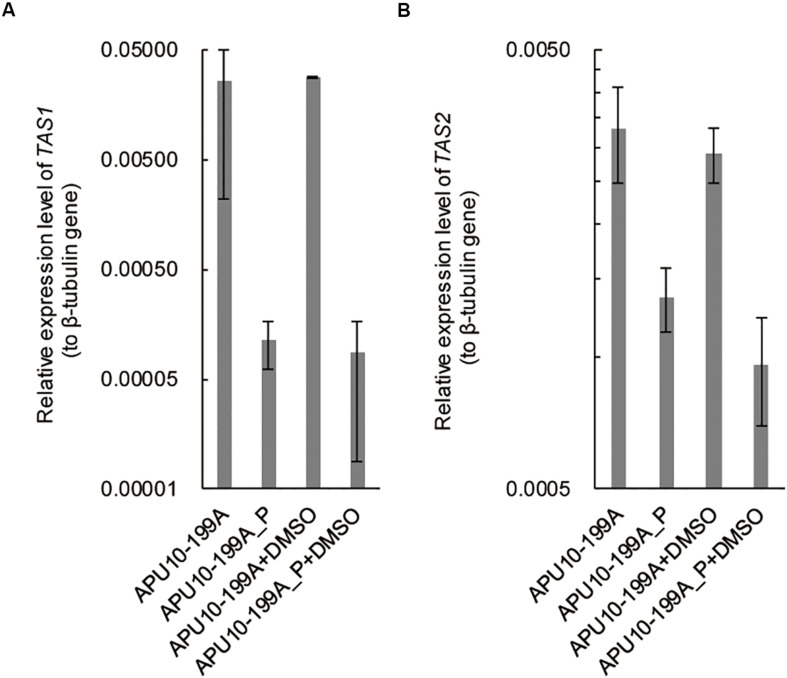
Expression levels of *TAS1* and *TAS2* in APU10-199A and APU10-199A_P. Each strain was cultured in SS medium, and the expression levels of **(A)** *TAS1* and **(B)** *TAS2* were determined by qRT-PCR. This experiment was performed on three independent biological replicates each containing three technical replicates. Error bars represent the standard deviation.

It has been reported that TeA is biosynthesized by a non-ribosomal peptide synthetase (NRPS) and PKS hybrid enzyme, TeA synthetase 1 (TAS1) in *M. oryzae* (Yun et al., 2015). The expression of *TAS1* is regulated by a Zn(II)_2_-Cys_6_-type transcription factor, TAS2 (Yun et al., 2017). To analyze the influence of the mycovirus(es) on the transcription of *TAS1* and *TAS2*, we determined the expression levels of these genes in APU10-199A and APU10-199A_P by qRT-PCR. The expression levels of both *TAS1* and *TAS2* were higher in APU10-199A than those in APU10-199A_P (Figure 3). These data suggested that a mycovirus enhances TeA production in *M*. *oryzae* by upregulating the transcription factor gene *TAS2*. Yun et al. (2015) also reported that 1% DMSO addition to culture induces TeA production in *M*. *oryzae*. Under our conditions, however, DMSO addition did not induce TeA production or enhance expression of *TAS1* and *TAS2* in APU10-199A or APU10-199A_P (Supplementary Figure S2 and Figure 3).

### Totivirus-Induced TeA Production Proved by Reinfection Experiment

**FIGURE 4:**
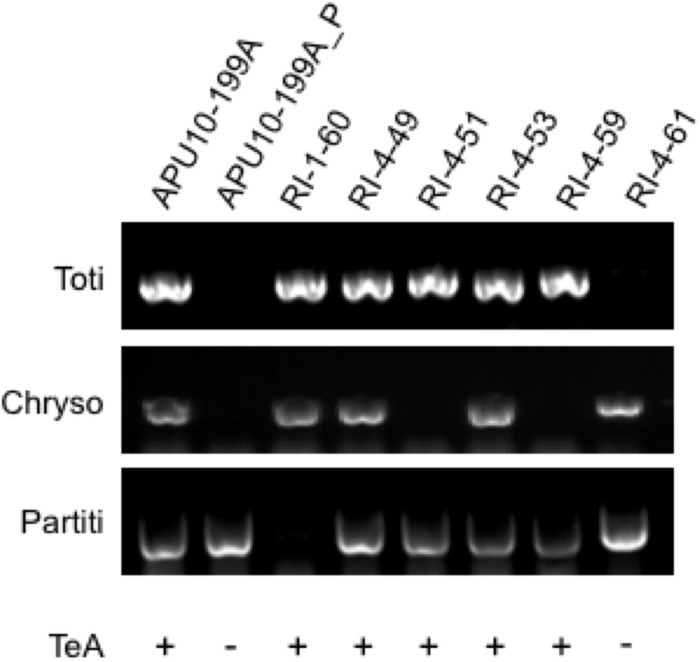
Virus infection profiles and tenuazonic acid production of the strains obtained by virus reinfection experiments. The presence of the totivirus, chrysovirus, and partitivirus was confirmed by RT-PCR using primers specific to each virus (for sequences of primers see Supplementary Table S2). Tenuazonic acid (TeA) productivity in each strain is shown by + or –.

The APU10-199A_P strain that did not produce TeA lacks the totivirus and chrysovirus. The next question is which mycovirus induces TeA production. To identify the TeA inducer, we established several re-infected virus strains derived from APU10-199A_P with the *hph* gene introduced as a selective marker through the hyphal anastomosis method (see section “Materials and Methods”). As a result, six reinfected (RI) strains (RI-1-60, RI-4-49, RI-4-51, RI-4-53, RI-4-59, and RI-4-61) were obtained (Supplementary Figure S3). The virus infection profiles of RI strains were checked by RT-PCR (Figure 4) and agarose gel electrophoresis of the dsRNA (Supplementary Figure S4). RI-4-49 and RI-4-53, infected with all three viruses, produced TeA (Figure 4; for the HPLC chart, see Supplementary Figure S5), which indicated that the introduction of the *hph* gene and reinfection manipulation do not affect the TeA production. RI-4-51 and RI-4-59, infected with the totivirus and the partitivirus, also produced TeA, which indicated that TeA production was not dependent on the chrysovirus. RI-4-61, infected with the chrysovirus and the partitivirus, did not produce TeA under our conditions. Thus, we concluded that TeA production depended on infection with a totivirus.

### Mycoviruses Affected Multiple SM Core Genes

For comprehensive understanding of the viral effects on secondary metabolism of *M*. *oryzae*, we performed RNA sequencing for the APU10-199A, APU10-199A_P, RI-4-49, and RI-4-51 strains. RI-4-49 is reinfected with both the totivirus and chrysovirus, while RI-4-51 is reinfected with only the former. The genome sequence of *M*. *oryzae* 70-15 (GCA_000002495.2) was searched for genes responsible for formation of core structures of SMs (hereinafter referred to as “core genes”) by antiSMASH 5.0 (Blin et al., 2019). Accordingly, 7 terpene synthases, 16 NRPSs, 21 PKSs, and 7 PKS-NRPS hybrid enzymes were found to be encoded in the genome, and the gene expression levels were determined from RNA-sequencing analysis (Table 1). First, we compared the expression levels of the 51 core genes between APU10-199A and APU10-199A_P to evaluate the influence of the totivirus and/or chrysovirus on the secondary metabolism of *M*. *oryzae*. We filtered the core genes differently expressed in APU10-199A and APU10-199A_P under the following conditions: the ratio of transcripts per million (TPM) > 10 or <0.1, and TPM > 1/10 mean TPM (>7.66). Three core genes, MGG_10671, MGG_14767, and *TAS1* (a terpene synthase plus two NRPSs) were differently expressed between APU10-199A and APU10-199A_P (Table 1). Whereas *TAS1* was highly expressed in APU10-199A, the other core genes, MGG_10671 and MGG_14767, were hardly expressed in APU10-199A. This suggested that the totivirus and/or chrysovirus inhibit transcription of the two core genes for secondary metabolism. This view was supported by the data that RI strains (RI-4-49 or RI-4-51) reinfected with both the totivirus and chrysovirus or only the totivirus showed lowered expressions of MGG_10671 and MGG_14767. In these RI strains, *TAS1* was as highly expressed as it was in APU10-199A. This result further confirmed that TeA was produced in a manner dependent on totivirus infection. Furthermore, the transcriptome data revealed that the expression level of *TAS2* in the RI strains was higher than that in APU10-199A_P (Table 1). Thus, we concluded that the totivirus enhances TeA production in *M*. *oryzae* by upregulating the transcription of the gene encoding TeA synthetase.

**TABLE 1:** Expression levels (TPM) of core genes for secondary metabolism determined by RNA-sequencing analysis.

Type	Name	Description	APU10-199A	APU10-199A_P	Ratio (199A/199A_P)	RI-4-49 (TCP)	RI-4-51 (TP)
	MGG_00758		**67.7**	**76.6**	0.884	**55.8**	**67.6**
	MGG_01949		0	1.11	0	0	0
	MGG_03432		0	0.0653	0	0	0.109
TS	MGG_03833		0.161	0.0576	2.80	0.106	0
	MGG_09239		**89.4**	**54.6**	1.64	**92.8**	**75.2**
	MGG_10671		0	**13.9**	0	0	2.01
	MGG_11702		**84.8**	**73.0**	1.16	**72.8**	**64.7**

	MGG_00022		0.0107	0.926	0.0116	0.0317	0.0670
	MGG_00385		**96.5**	**165**	0.585	**72.2**	**112**
	MGG_02351		0.197	0.176	1.12	0.187	0.420
	MGG_02611		**89.0**	**118**	0.754	**93.3**	**90.7**
	MGG_03290		**27.2**	**50.6**	0.538	**26.2**	**38.0**
	MGG_03401		**52.1**	**43.9**	1.19	**38.6**	**41.8**
	MGG_03422		0	0	-	0.0379	0
NRPS	MGG_05491		0.0407	0.261	0.156	0	0
	MGG_07803 (*TAS1*)	TeA synthetase	**163**	0.134	1220	**47.9**	**107**
	MGG_07858		0	0.0292	0	0	0
	MGG_12175	ferricrocin synthetase *SSM1*(Hof et al., 2007)	**118**	**97.4**	1.21	**73.5**	**73.7**
	MGG_14767		0.0582	**270**	0.000216	1.31	3.93
	MGG_14967		0.200	0.211	0.948	0.188	0.101
	MGG_15248		**9.23**	**7.96**	1.16	**8.31**	**8.32**
	MGG_16971		2.96	2.73	1.08	4.92	4.00
	MGG_17746		0.198	7.62	0.0260	0.893	1.05

	MGG_00233		0.127	3.04	0.0418	0.216	0.359
	MGG_00241		0	0	-	0.0399	0
	MGG_00428		**15.3**	**15.6**	0.981	**18.0**	**14.6**
	MGG_04775		4.80	5.53	0.868	7.40	**9.80**
	MGG_05589		0.790	0.144	5.49	0.734	0.242
	MGG_06254		**11.5**	**11.9**	0.966	**11.8**	**13.2**
	MGG_07219	melanin synthase *ALB1* (Chumley and Valent, 1990)	0.234	0.125	1.87	0.116	0.0525
	MGG_08236		0.0332	0.142	0.234	0.197	0.164
	MGG_08281		0.0700	1.04	0.0673	0.0923	0.0209
	MGG_08285		**8.62**	6.18	1.39	**8.35**	**14.1**
PKS	MGG_09645		0.0204	0	-	0	0
	MGG_10011		0.0564	2.64	0.0214	0	0.0169
	MGG_10912	pyriculol synthase *MoPKS19* (Jacob et al., 2017)	0.932	2.80	0.333	0.572	1.09
	MGG_11638		2.23	0.977	2.28	2.71	2.00
	MGG_12214		**16.7**	**14.6**	1.14	**16.0**	**13.7**
	MGG_12613		2.03	0.948	2.14	1.40	1.25
	MGG_13591		0	0.0200	0	0	0
	MGG_13767		2.35	0.174	13.5	0.843	1.07
	MGG_14831		0.0336	1.17	0.0287	0.598	0.346
	MGG_14945		0	0.0210	0	0	0
	MGG_15100		**172**	**133**	1.29	**170**	**991**

	MGG_03810		0.0306	6.02	0.00508	0.222	0.571
	MGG_09589		0.412	0.913	0.451	0.617	0.714
	MGG_12447	avirulence gene *ACE1* (Collemare et al., 2008)	0	0.392	0	0	0.0193
PKS-NRPS	MGG_14897		1.02	1.32	0.773	0.961	1.50
	MGG_14943		0.688	4.86	0.142	0.735	2.58
	MGG_15097		0	1.17	0	0	0
	MGG_15272		2.47	1.50	1.65	3.50	**9.08**

Transcription factor	MGG_07800 (*TAS2*)	transcription factor for TeA biosynthesis	**30.3**	**8.04**	3.77	**30.7**	**41.1**

*The levels over mean TPM × 0.1 (7.66) are shown in bold.*

## Discussion

Fungi have the potential to produce various SMs with biological activities. While fungal SMs are resources for potent therapeutic agents, some fungal SMs are toxic and harmful to humans or animals. Thus, control of fungal secondary metabolism is needed for improvement of public health. To date, some knowledge on control of fungal SM production by mycoviruses has accumulated. Some mycoviruses upregulate (Okada et al., 2018; Nerva et al., 2019) or downregulate (Schmidt et al., 1986; Chu et al., 2002) mycotoxin production. However, these studies regarding SM production were based on comparison between the strains with and without viral infection. In the present study, we used reinfected strains to prove that mycovirus infection affects SM production in *M*. *oryzae*. Furthermore, the effect of mycovirus on biosynthetic gene expression was observed in fungi, probably for the first time. These achievements pave the way for more extensive screening for fungal bioactive compounds by taking advantage of mycovirus.

The totivirus has a monosegmented dsRNA genome (5.2 kb) encompassing two open reading frames (ORFs), each of which encodes a coat protein and a RdRp. Although the detailed molecular mechanism remains unclear, either or both of the two ORFs are possibly responsible for TeA induction. Further research is needed to determine the exact factor encoded in the genome of the totivirus that induces TeA production. The direct target that the viral factor interacts with and the signaling pathway are also still unknown. Elucidation of the signaling pathway that triggers *TAS2* activation is the main goal of our follow-up studies. In addition to induction of TeA, production of several SMs was likely to be suppressed by the chrysovirus or totivirus (Figure 1). Notably, different culture conditions, such as media and agitation speed, led to different mycovirus effects on SM production. This may indicate that viral effects are exerted on multiple aspects of cellular response. In this study, we could not obtain the strain that carries totivirus alone. The possibility that combination of totivirus and partitivirus or chrysovirus is required for the TeA induction cannot be ruled out at the moment. Further research is needed to clarify this question.

TeA is one of the compounds that have been identified from fungal cultures in early studies, and a variety of biological activities, including antibacterial, antiviral, and phytotoxic activities, have been reported (Miller et al., 1963; Gitterman, 1965; Chen et al., 2010). In a study of *Alternaria* species, it was proposed that TeA plays a role in fungal infection as a virulence factor (Kang et al., 2017). So far, the function of TeA in the *M. oryzae* infection process remains unclear despite its bioactivity. Yun et al. (2015) recently discovered the TeA biosynthetic gene in *M. oryzae* and reported two TeA-inducing conditions, 1% DMSO addition and deletion of an osmo-sensory MAPK-encoding gene, *OSM1*. However, there are only a few reports on TeA production in *M. oryzae*, suggesting that the TeA-producing strain is rare or that TeA is produced under limited conditions. This is because our finding that totivirus-triggered TeA production in the fungus is of great value for investigating rarely expressed fungal SMs. Viral effects induced by an endogenous genetic factor without any chemical addition or gene manipulation would allow us to activate unstudied and silent fungal secondary metabolism. Development of a viral transfer method is focus of future work.

Comprehensive sequencing of fungal genomes has revealed that fungi have many more gene clusters for SMs than the quantity estimated from the number of known SMs (Khaldi et al., 2010; Sanchez et al., 2012). However, only a few of the gene clusters are expressed under normal laboratory conditions (Brakhage and Schroeckh, 2011). In our case, out of 51 core genes encoded in the genome of *M*. *oryzae*, 17 genes (33.3%) were expressed in either APU10-199A or APU10-199A_P with a threshold of TPM: mean TPM × 0.1 (Table 1). Because of the potential of fungal cryptic gene clusters as a resource for new bioactive compounds, several strategies have been employed to express cryptic genes. One of the major strategies is co-culturing of a fungus and another microorganism. For example, Schroeckh et al. (2009) revealed that interaction between *Aspergillus nidulans* and a streptomycete induces production of several fungal SMs through activation of the orselinic acid biosynthetic gene cluster. Another strategy is addition of chemical elicitors. Williams et al. (2008) discovered new compounds from two fungi using DNA methyltransferase and histone deacetylase inhibitors as an elicitor of secondary metabolism. The other strategy that increasingly draws attention from chemists who are familiar with fungal genetic manipulation is a heterologous expression system for fungal SM gene(s) (Kennedy et al., 1999; Heneghan et al., 2010; Itoh et al., 2010). Although these are powerful tools for discovery of new natural products and elucidation of biosynthetic pathways, there is no versatile method that can be applied to any gene cluster for SMs. Thus, it is important to establish a variety of alternatives for activating silent biosynthetic gene clusters. In this report, we discovered activation of the TeA biosynthetic gene by a totivirus. Some mycoviruses can be transmitted into fungal cells by hyphal anastomosis, protoplast fusion (Lee et al., 2011), or electroporation (Moleleki et al., 2003). This underscores the potential of mycoviruses as an inducer of fungal secondary metabolism.

## Data Availability Statement

The datasets generated for this study can be found in the NCBI or DDBJ DRA under accession number PRJDB9423.

## Author Contributions

DH conceived, supervised this study, and wrote the manuscript. SU co-designed and co-supervised this study. AN carried out the analyses and wrote the manuscript. RS performed NMR analysis. SF and HM were involved in preparation of the experimental materials. All authors read and approved the final version of the manuscript.

## Conflict of Interest

The authors declare that the research was conducted in the absence of any commercial or financial relationships that could be construed as a potential conflict of interest.
