# Supplementary material for: Mycovirus-Induced Tenuazonic Acid Production in a Rice Blast Fungus Magnaporthe oryzae
Source: Front Microbiol. 2020 Jul 17;11:1641. doi: 10.3389/fmicb.2020.01641 (PMC7379127; doi:10.3389/fmicb.2020.01641)
Supplement: Supplementary file 1 [file Data_Sheet_1.docx]

**SUPPLEMENTARY DATA**

**Mycovirus-induced tenuazonic acid production in a rice blast fungus *Magnaporthe* *oryzae***

Akihiro Ninomiya,^1^ Syun-ichi Urayama,^1, 2^ Rei Suo,^3^ Shiro Itoi,^3^ Shin-ichi Fuji,^4^ Hiromitsu Moriyama,^5^ Daisuke Hagiwara^1, 2*^

^1^Faculty of Life and Environmental Sciences, University of Tsukuba, 1-1-1 Tennodai, Tsukuba, Ibaraki 305-8577, Japan.

^2^Microbiology Research Center for Sustainability, University of Tsukuba, 1-1-1 Tennodai, Tsukuba, Ibaraki 305-8577, Japan.

^3^College of Bioresource Sciences, Nihon University, 1866 Kameino, Fujisawa, Kanagawa 252-0880, Japan.

^4^Faculty of Bioresource Sciences, Akita Prefectural University, 241-386 Kaidobata Nishi, Nakano, Shimoshinjo, Akita 010-0195, Japan.

^5^Department of Applied Biological Sciences, Tokyo University of Agriculture and Technology, 3-5-8 Saiwaicho, Fuchu, Tokyo 183-8509, Japan.

*Corresponding author

+81-29-853-2672, hagiwara.daisuke.gb@u.tsukuba.ac.jp

**Figures**

Figure S1. HRESIMS spectrum of tenuazonic acid (**1**).

Figure S2. Secondary metabolite profiles of APU10-199A and APU10-199A_P cultured in soy sauce-sucrose medium containing 1% dimethyl sulfoxide.

Figure S3. Colonies of the strains used in this study grown on PDA.

Figure S4. Secondary metabolite profiles of RI strains.

Figure S5. Electrophoresis of total double-stranded RNAs isolated from the strains used in this study.

**Tables**

Table S1. ^1^H and ^13^C NMR data (CD_3_OD) for tenuazonic acid (**1**).

Table S2. Sequences of primers used in this study.


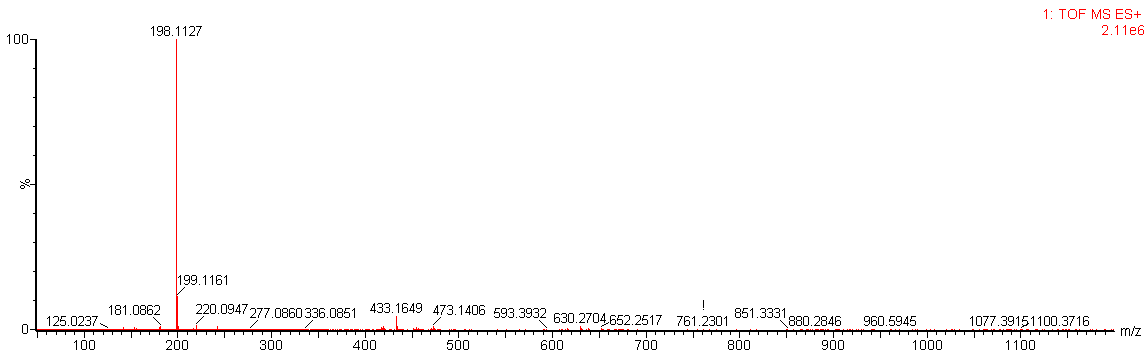


Figure S1. HRESIMS spectrum of tenuazonic acid (**1**).


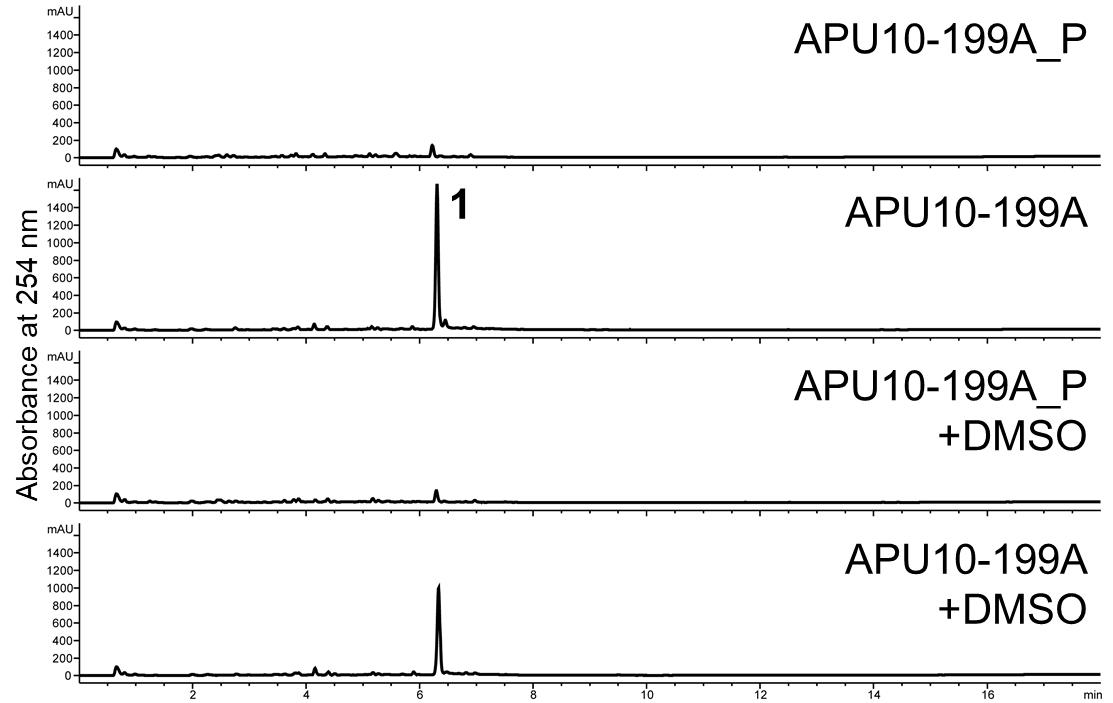


Figure S2. Secondary metabolite profiles of APU10-199A and APU10-199A_P cultured in soy sauce-sucrose medium containing 1% dimethyl sulfoxide. Production of tenuazonic acid in APU10-199A_P was not induced by addition of dimethyl sulfoxide. This data shows a representative profile from three independent culture experiments.


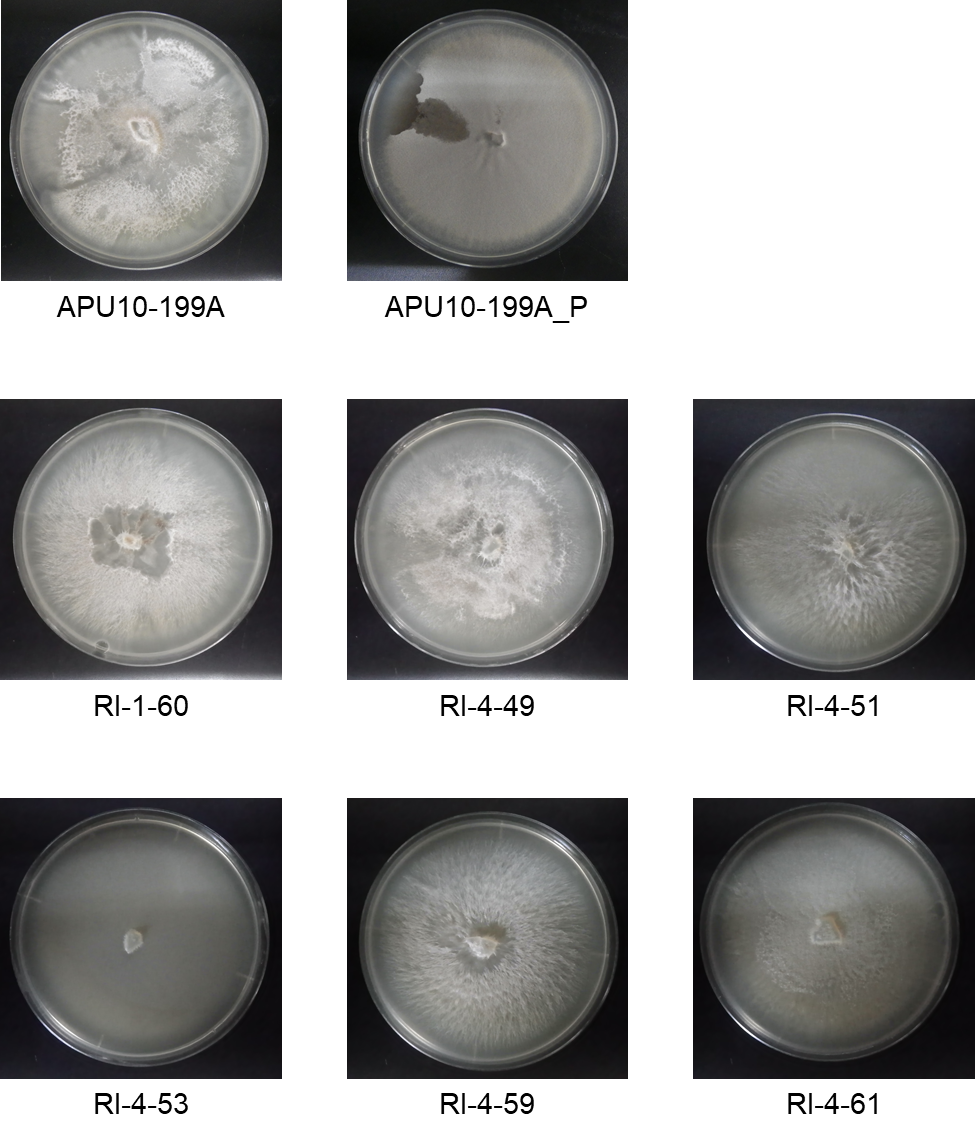


Figure S3. Colonies of the strains used in this study grown on PDA.


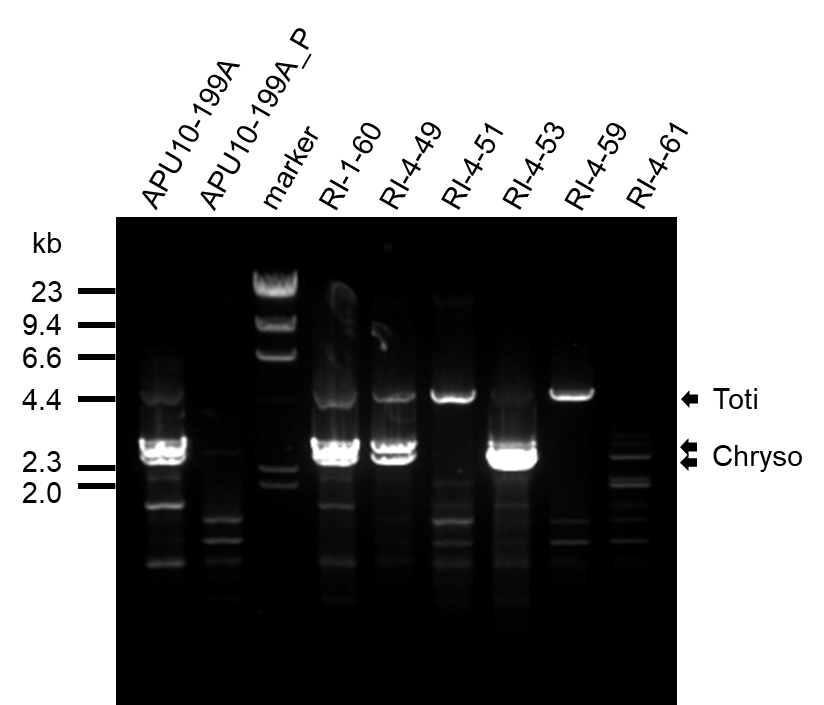


Figure S4. Electrophoresis of total double-stranded RNAs isolated from the strains used in this study.


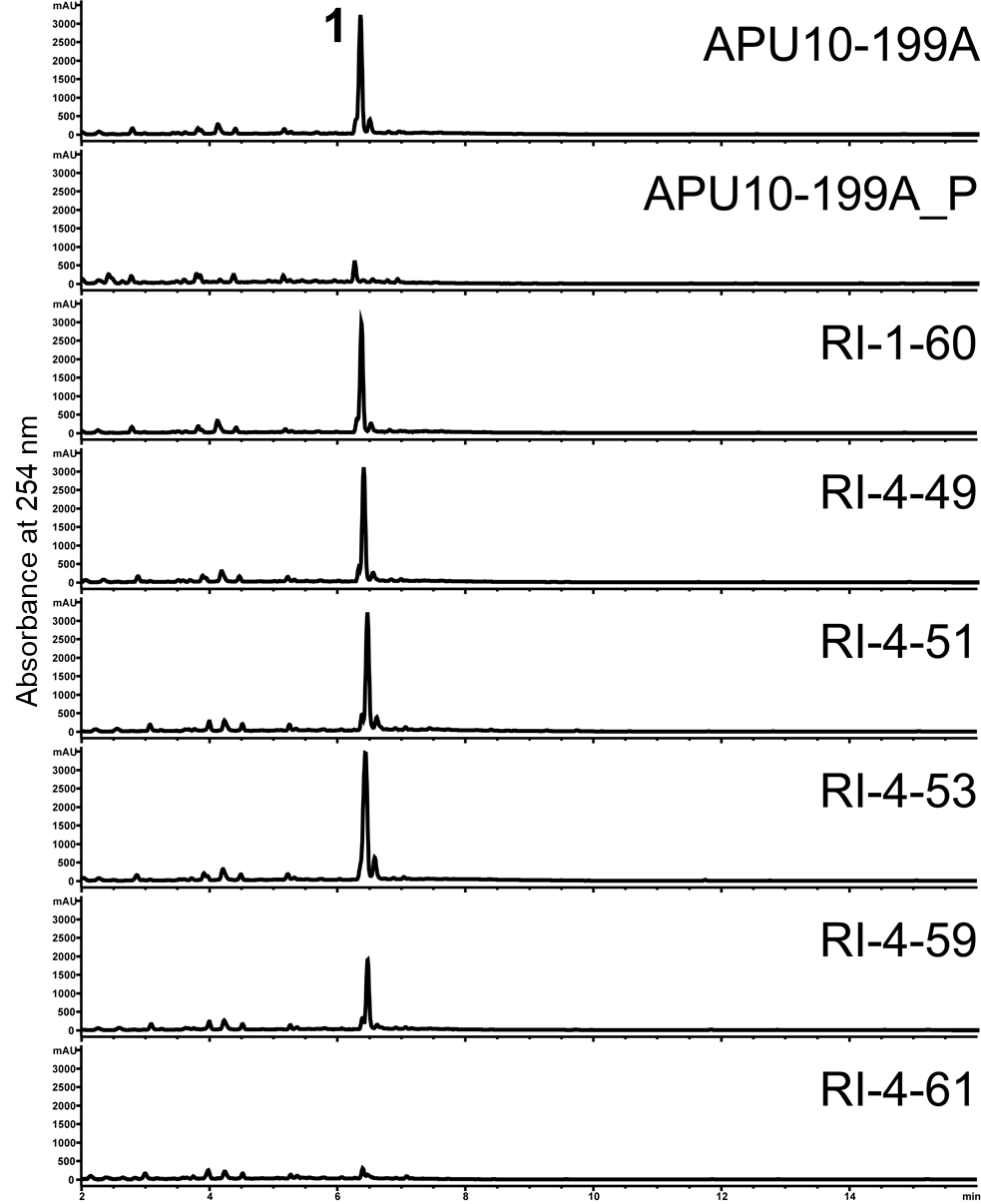


Figure S5. Secondary metabolite profiles of RI strains.

Table S1. NMR data for tenuazonic acid (**1**) in CD_3_OD.

Table S2. Sequences of primers used in this study.


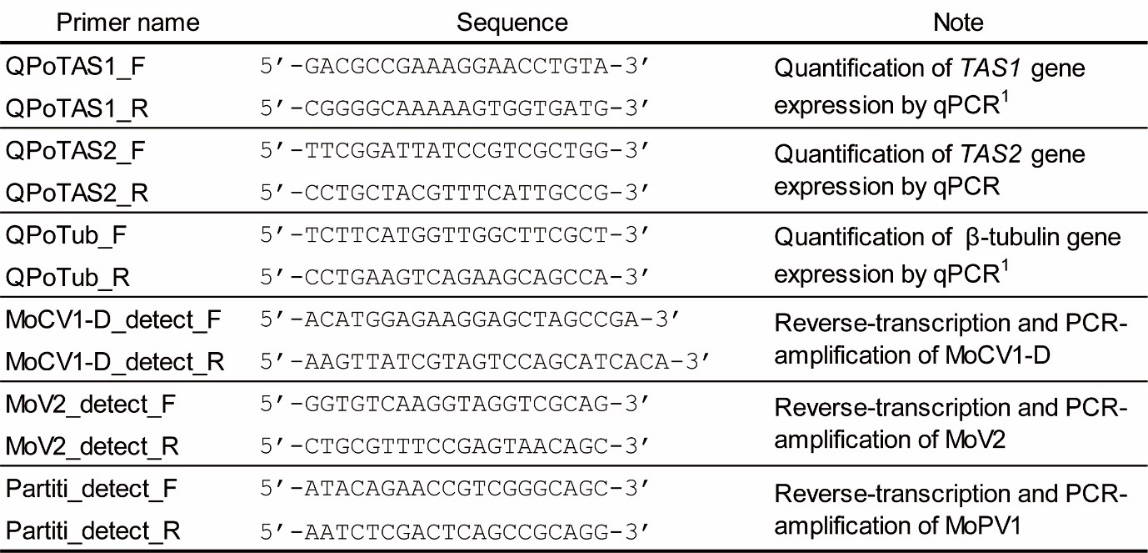


Reference 1: Yun C-S, Motoyama T, Osada H. Biosynthesis of the mycotoxin tenuazonic acid by a fungal NRPS-PKS hybrid enzyme. *Nat* *Commun* 2015;**6**:8758.
